# Occurrence and Distribution of Arsenic, Antimony and Selenium in Shallow Groundwater Systems of Ibadan Metropolis, Southwestern Nigerian

**DOI:** 10.5696/2156-9614-7-13.32

**Published:** 2017-03-29

**Authors:** Effiong Ukorebi Etim

**Affiliations:** Department of Chemistry, University of Ibadan, Ibadan, Nigeria

**Keywords:** groundwater, risk assessment, arsenic, antimony, selenium

## Abstract

**Background.:**

Arsenic, antimony and selenium contamination of groundwater is of great concern due to the potential detrimental effects to human health.

**Objectives.:**

This study investigates the occurrence and distribution of arsenic, antimony and selenium in the shallow groundwater system of Ibadan metropolis, southwestern Nigeria.

**Methods.:**

A total of 210 groundwater samples were collected from 35 shallow wells (3.15–7.86 m) within the residential, commercial, industrial and agricultural areas of the metropolis during the dry and wet seasons. The average daily dose intake (ADD), hazard quotient (HQ) and hazard index (HI) of arsenic, antimony and selenium exposure in groundwater were calculated from these four studied areas for children and adults.

**Results.:**

Average concentrations of arsenic, antimony and selenium in groundwater ranged between 2.17±3.49 to 33.8±37.2 μg/L, 13.5±15.0 to 33.2±36.8 μg/L and 7.33±6.22 to 46.3±22.4 μg/L, respectively. A corresponding analysis relay plot showed the order of occurrence of these trace metals in groundwater to be antimony>selenium>arsenic. The principal component analysis biplot showed that arsenic, antimony and selenium were fairly distributed in all of the study areas, suggesting the influence of geogenic factors. A total of 74.3% of sampling locations had antimony levels slightly above the World Health Organization (WHO) safe limit of 20 μg/L. Statistical t testing (0.05 confidence limit) showed a significant difference in seasonal levels of groundwater antimony concentration, with the dry season recording significantly higher levels with 100% of samples exceeding WHO safe limits. The chemical of highest potential human health concern is antimony, with a non-carcinogenic HQ risk factor >2 for both age groups. The overall non-carcinogenic HI was highest in the commercial area, 4.1989 for adults and 5.2487 for children.

**Conclusions.:**

Antimony in groundwater within the Ibadan metropolis raises health concerns and a concerted effort is needed to identify its sources to avoid the risk of antimony toxicity.

## Introduction

Arsenic and antimony are ubiquitous metalloids widely distributed in the earth's crust and most commonly found in natural groundwater aquifers.^1^,^2^,^3^ Concentrations in groundwater are often associated with geological formations such as igneous and sedimentary rocks, and ores of different metals.^4^,^5^,^6^ They are introduced naturally into groundwater through weathering processes of geogenic mineral rocks, leaching of wet deposition and microbial activities.^7^,^8^ Contamination can also be caused by anthropogenic activities such as mining, groundwater abstraction, industrial effluent sources and pesticide application in agricultural fields.^9^,^10^ More often, it is natural processes that play a dominant role in arsenic mobility in groundwater, while antimony pollution originates mainly from mining and industrial emissions.^5^,^10^,^11^,^12^ Arsenic and antimony are mostly found in inorganic forms in groundwater as trivalent arsenic [As (III)] or pentavalent arsenic [As (V)], trivalent antimony [Sb (III) or pentavalent antimony [Sb (IV)].

Their oxidation state is guided by changes in the redox conditions of the aquatic environment. Trivalent species have been reported to be more toxic than pentavalent forms and information on their distribution and transportation in the environment is very limited.^13^,^14^ Arsenic and antimony have no known biological function and are extremely toxic. Both metalloids are clastogenic in the trivalent state, and have carcinogenic potential.^4^,^15^,^16^ Antimony is considered a priority pollutant of interest by the United States Environmental Protection Agency (US EPA) and the European Union (EU)^17^,^18^ and are currently on the list of hazardous substances under the Basel Convention concerning the restriction of trans-boundary movement of hazardous chemicals.^19^ Concentrations of arsenic and antimony in groundwater are usually very low, as they depend on the location and proximity of pollution sources.^20^,^21^,^22^ In some instances, concentrations can reach far in excess of statutory drinking water regulatory limits and thus are a potentially severe public health threat. Typical background concentrations of arsenic and antimony in groundwater are often less than the 10 μg/L and 20 μg/L limit of the World Health Organization (WHO),^23^,^24^,^25^ although incidences of elevated concentrations have been reported in many countries, such as Cambodia, China, Mexico, Argentina, India, Bangladesh, Croatia and Southern Vietnam.^1^,^26^,^27^,^28^,^29^,^30^,^31^,^32^

Selenium is an essential micronutrient for organisms and humans and is also found in groundwater. It is essential at a limited daily intake, but is toxic at elevated amounts and is therefore classified as an ‘essential toxicant’.^6^,^33^ The presence of selenium in groundwater is primarily linked to mining activities and industrial emissions. In groundwater, selenium exists in the dissolved forms of Se (VI), Se (IV) and Se (-II). Selenium (IV) is more toxic due to its higher bioavailability.^34^ Selenium is known to be carcinogenic at higher concentrations,^33^ but at much lower levels has anti-carcinogenic properties which prevent arsenic toxic effects.^35^,^36^,^37^ Less is known about its speciation, distribution and transformation in groundwater.^38^,^39^ Selenium levels in groundwater are usually very low, although levels can be significant in areas of pollution.^39^,^40^

Of the various routes of exposure of arsenic, antimony and selenium, consumption of contaminated drinking water poses the greatest risk to public health. Industrial emissions and mining activities are other possible routes of exposure which may pose significant local risk. This has necessitated studies characterizing their occurrence and distribution in groundwater in many countries. In Nigeria, reliable data on arsenic, antimony and selenium concentrations in groundwater are scant, and often limited to very small areas.^41^,^42^ Examination of groundwater quality in large cities like Lagos and Ibadan has largely centered on microbial and physico-chemical characteristics and heavy metals such as lead (Pb), chromium (Cr), cadmium (Cd), cobalt (Co), nickel (Ni), zinc (Zn) and iron (Fe).^43^,^44^,^45^ Arsenic, antimony and selenium are often not on the list of constituents routinely analyzed in groundwater due to limited analytical instrumentation. The current study investigates the occurrence and distribution of arsenic, antimony and selenium in groundwater in Ibadan metropolis, southwestern Nigeria. A standard risk assessment model was used to estimate the non-carcinogenic effects of the metals on adults and children in the study area.

**Abbreviations**
*HI*: Hazard Index
*HQ*: Hazard Quotient
*US EPA*: United States Environmental Protection Agency
*WHO*: World Health Organization

## Methods

### Study Area

**Figure 1:**
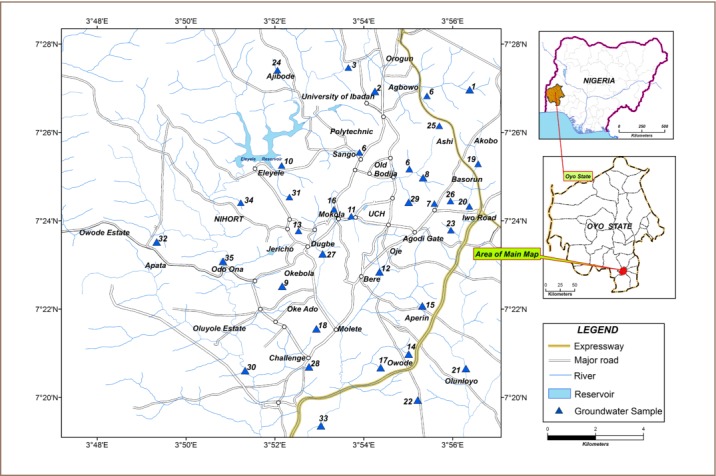
Map of Ibadan metropolis showing groundwater sample locations

Ibadan metropolis is located in the southwestern part of Nigeria, the capital city of Oyo State, and consists of five local government areas (*Figure 1*). It has an area of about 1350 km^2^ and lies between 7^°^ 28′N and 3° 48′E, with an estimated population of 1,338,659.^46^ The city metropolis is a mix of residential, commercial, industrial and agricultural areas and relies solely on groundwater for domestic, industrial and agricultural purposes. The area is underlined by the igneous and metamorphic rocks of the Precambrian basement complex consisting of mainly granite-gneiss, biotite-gneiss, pegmatite, migmatite, schists and quartzite.^47^ Groundwater occurs in the shallow weathered zones, as well as deeper fractured zones. The current study is limited to the shallow weathered portion of the aquifer containing fresh potable groundwater.

### Sampling Collection and Preservation

Groundwater samples were collected from 35 open dug wells during the months of January, February and March (dry season) and May, June and July (wet season) in 2016. The sampling wells were chosen to represent residential (24), commercial (5), industrial (3) and agricultural (2) areas. A total of 210 groundwater samples were collected during the study period. The depth of sampled wells ranged between 3.15 to 7.86 m below ground level. Groundwater samples were separately collected for physicochemical analysis in pre-cleaned 1 L polytetrafluoroethylene bottles. Samples for arsenic, antimony and selenium determination were collected using pre-cleaned 500 mL polytetrafluoroethylene bottles, and acidified to pH < 2 using nitric acid. Each sample container was rinsed three times with water from the well before filling in order to condition the container with the groundwater sample. All samples were transported to the laboratory under low-temperature conditions (4° C) in ice-boxes and analyses were performed immediately. Field blank samples were prepared and treated in the same manner as study samples.

### Analytical Methods

Deionized water was used throughout the analysis and chemicals were of AnalaR grade. The samples were analyzed for pH and total dissolved solids by the electrometric method (Jenway 3510 pH meter and Philips ECscan 40 conductivity tester); alkalinity, chloride, total hardness, calcium and magnesium by the titrimetric method; chemical oxygen demand by the wet oxidation method; biochemical oxygen demand by the dilution method; and nitrate, phosphate, sulphate and ammonia by the spectrophotometric method using standard procedures of the Society for Analytical Chemistry^48^ and the American Public Health Association, American Water Works Association and Water Pollution Control Federation.^49^ Arsenic, antimony and selenium were determined in the samples by inductively coupled plasma optical emission spectrometry (ICP-OES) (Perkin Elmer, Elan 4000). The accuracy and precision of ICP-OES measurements were approximately 1 and 3%, respectively. Analysis of six field blanks showed no evidence of contamination. Recovery studies carried out by spiking five groundwater samples with standard arsenic, antimony and selenium ranged between 95.7–98.5% for arsenic, 94.2–98.9% for antimony and 93.5–97.2% for selenium.

### Risk Assessment Evaluation

The risk of arsenic, antimony and selenium levels in groundwater to adults and children within the different study areas [residential, commercial, industrial and agricultural] was evaluated based on the risk assessment techniques proposed by the US EPA.^50^,^51^ The average amount of daily drinking water consumption was considered to be 2.0 L for adults and 1.0 L for children, and the average body weight was considered to be 50 kg for adults (20–50) and 20 kg for children (3–20).^50^,^51^ Therefore, the lifetime average daily dose per person was calculated using Equation 1.

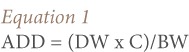



Where, ADD is the average daily dose consumed with drinking water, DW is the average amount of water consumed every day (L), C is the concentration of the chemical substance (mg/L) and BW is average body weight (kg).

The hazard quotient (HQ), the ratio of the potential exposure to a substance and the level at which no adverse effects are expected, was calculated using Equation 2 to estimate the non-carcinogenic risk of arsenic, antimony and selenium in drinking water. If the HQ is calculated to be less than 1, then no adverse health effects are expected as a result of exposure. If the HQ is greater than 1, then adverse health effects are possible.^52^

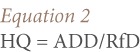



Where, ADD is the exposure dose obtained from Equation 1 and RfD is the reference dose of the contaminant. RfD represents a dose below which toxicity does not affect humans (including sensitive subgroups) during a lifetime. The recommended RfD values are 0.0003 mg/kg/day for arsenic, 0.0004 mg/kg/day for antimony and 0.005 mg/kg/day for selenium.^53^

The overall non-carcinogenic risk posed by the three metals was assessed by adding up the HQ of each metal and expressed as Hazard Index (HI) by Equation 3.

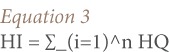



### Statistical Data Analysis

Correspondence analysis was performed on the metal data set to show the order of prevalence and occurrence. Principal component analysis was established to identify the distribution of metals within the sampling areas. The degree of significance of the metal data was carried out by analysis of variance.

## Results

### Hydrochemistry of Groundwater

The general hydrochemistry of groundwater in Ibadan metropolis is given in Table 1. The pH value indicates acidic conditions with an average value of 5.53±0.28 (5.21–6.01). Groundwater pH value, alkalinity, total hardness, calcium, magnesium, total dissolved solids, turbidity, chemical oxygen demand, biochemical oxygen demand, nitrate, sulphate and phosphate levels from this study were relatively consistent with typical values previously reported for the metropolis.^44^ No pattern of spatial distribution was determined for any of the above variables. Except for pH, most of the parameters in the groundwater supply were within the allowed limits for drinking water set by the WHO,^23^ US EPA,^54^ and the Federal Ministry of the Environment,^55^ as shown in Table 1. The low pH values may be a result of the production of CO_2_ from microbial respiration, and may enhance dissolution of trace elements from mineral rock.^56^

**Table 1:** General Hydrochemistry of Sampled Groundwater

**Sample Number**	**Parameter**	**Mean±SD**	**Range**	**WHO, 2011**^23^	**US EPA, 1999**^54^	**Federal Ministry of the Environment, Nigeria,1993**^55^
1	pH	5.53±0.28	5.21–6.01	6.5–8.5	6.5–8.5	6.5–8.5
2	Alkalinity (mgCO_3_/L)	257±104	163–426	-	-	-
3	Total hardness (mg/L)	192±102	98.5–374	500	-	200
4	Calcium (mg/L)	70.7±37.5	34.6–136	200	-	-
5	Magnesium (mg/L)	6.25±3.19	3.96–12.6	-	-	-
6	Total dissolved solids (mg/L)	358±160	173–635	<600	500	500
7	Turbidity (FTU)	4.19±0.94	2.84–5.15	<1	-	-
8	Chemical oxygen demand (mg/L)	61.2±9.17	48.6–74.3	-	-	-
9	Biochemical oxygen demand (mg/L)	2.92±1.17	1.75–4.65	-	-	0
10	Nitrate (mg/L)	4.26±1.46	2.85–6.87	50	10	10
11	Sulphate (mg/L)	40.7±23.3	19.4–83.7	500	250	500
12	Phosphate (mg/L)	0.79±0.44	0.34–1.22	-	-	<5

### Total Arsenic, Antimony and Selenium Concentrations in Groundwater

Table 2 shows the arsenic, antimony and selenium levels determined in groundwater. Antinomy and selenium levels were higher in groundwater than arsenic. Average arsenic concentration in groundwater ranged between 2.17±3.49 to 33.8±37.2 μg/L with a general average of 2.32 μg/L within the metropolis. Typical detection levels (i.e above the instrument detection limit, only found in the dry season) were obtained in only 17% of samples collected. Antimony was detected in groundwater samples in all locations with an average range of 13.5±15.0 to 33.2±36.8 μg/L. The general average antimony level in groundwater within the metropolis was 24.9±26.2 μg/L. The average concentrations of selenium ranged between 7.33±6.22 to 46.3±22.4 μg/L with a typical average of 22.5±12.5 μg/L. Table 3 shows average seasonal arsenic, antimony and selenium levels in groundwater. Arsenic was not detected during the wet season (0 μg/L), while only 17% of total sampled groundwater showed arsenic (4.33±4.0 to 67.6±4.7 μg/L) in the dry season. Two of the sampling locations within the commercial areas recorded very high arsenic levels (67.6±4.7 and 50.7±7.1 μg/L). A high level of antimony (27.0±3.6 to 70.0±7.9 μg/L) was detected in all of the groundwater samples during the dry season period. Selenium levels were slightly higher in the dry season (0.026 to 66.0±8.2 μg/L) than the wet season (1.33±2.3 to 35.3±2.1 μg/L).

**Table 2:** Arsenic, Antimony and Selenium Levels (μg/L) in Groundwater

**Codes**	**Arsenic**		**Antimony**		**Selenium**	
	Mean±SD	Range	Mean±SD	Range	Mean±SD	Range
GW1^*^	0	0	19.0±21.2	(0–45.0)	21.2±19.6	(2.0–43.0)
GW2^*^	0	0	28.0±15.3	(10.0–46.0)	20.7±19.3	(1.0–32.0)
GW3^*^	0	0	25.8±29.8	(0–67.0)	12.8±11.4	(0–26.0)
GW4^*^	6.67±8.48	(0–21.0)	28.8±25.2	(0–66.0)	28.7±21.1	(4.0–53.0)
GW5^*^	0	0	21.8±20.5	(0–44.0)	16.7±14.7	(2.0–34.0)
GW6^*^	2.17±3.49	(0–8.0)	28.8±31.7	(0–63.0)	11.3±7.9	(3.0–21.0)
GW7^*^	0	0	13.5±15.1	(0–32.0)	8.50±6.5	(0–14.0)
GW 8^*^	0	0	19.7±25.0	(0–61.0)	7.33±6.22	(0–15.0)
GW 9^*^	0	0	20.8±23.8	(0–54.0)	44.7±16.8	(26.0–74.0)
GW 10^*^	0	0	18.8±22.6	(0–54.0)	31.7±4.0	(24.0–36.0)
GW 11^*^	0	0	28.3±24.6	(0–65.0)	20.5±9.7	(8.0–33.0)
GW 12^*^	0	0	33.2±36.8	(0–76.0)	30.3±25.5	(4.0–58.0)
GW 13^*^	0	0	27.5±30.6	(0–63.0)	14.3±7.9	(4.0–23.0)
GW 14^*^	0	0	15.2±19.2	(0–47.0)	16.5±6.2	(9.0–25.0)
GW 15^*^	0	0	32.8±23.5	(5.0–56.0)	29.0±10.1	(17.0–43.0)
GW 16^*^	6.67±7.47	(0–16.0)	38.7±34.9	(10.0–76.0)	9.17±5.2	(0–16.0)
GW 17^*^	0	0	24.0±23.1	(0–52.0)	7.83±5.1	(0–14.0)
GW 18^*^	0	0	20.2±23.3	(0–44.0)	46.3±22.4	(23.0–73.0)
GW 19^*^	0	0	13.5±15.0	(0–31.0)	11.8±6.5	(10.0–16.0)
GW 20^*^	0	0	23.2±26.0	(0–54.0)	20.2±10.9	(6.0–33.0)
GW 21^*^	0	0	19.5±23.1	(0–55.0)	37.5±20.4	16.0–64.0)
GW 22^*^	0	0	29.2±32.1	(0–62.0)	41.5±23.2	(17.0–66.0)
GW 23^*^	0	0	29.8±32.8	(0–63.0)	20.2±12.7	(3.0–35.0)
GW 24^*^	0	0	29.0±31.9	(0–63.0)	29.7±13.9	(16.0–44.0)
GW 25^**^	33.8±37.2	(0–73.0)	28.3±31.2	(0–62.0)	31.3±8.1	(21.0–41.0)
GW 26^**^	25.3±28.1	(0–57.0)	28.5±31.5	(0–64.0)	44.5±10.8	(33.0–58.0)
GW 27^**^	0	0	18.2±20.6	(0–44.0)	24.5±17.8	(7.0–46.0)
GW 28^**^	0	0	26.3±29.2	(0–61.0)	25.7±20.7	(5.0–53.0)
GW 29^**^	0	0	17.8±20.0	(0–43.0)	27.3±17.9	(8.0–51-0)
GW 30^***^	0	0	26.3±28.9	(0–52.0)	9.50±9.61	(0–23.0)
GW 31^***^	0	0	26.8±29.5	(0–56.0)	16.5±12.5	(2.0–33.0)
GW 32^***^	6.67±8.48	(0.21.0)	30.3±33.4	(0–64.0)	16.2±11.8	(0–32.0)
GW 33^***^	0	0	24.2±26.6	(0–52.0)	11.2±10.1	(0–23.0)
GW 34^****^	0	0	23.3±25.7	(0–51.0)	23.2±5.3	(17.0–31.0)
GW 35^****^	0	0	30.8±33.9	(0–66.0)	18.5±5.4	(13.0–27.0)

^*^Residential areas;

^**^Commercial areas:

^***^Industrial areas;

^****^Agricultural areas

**Table 3:** Average Seasonal Metal Levels (μg/L) in Groundwater

**Codes**	**Wet Season**				**Dry Season**	
	**Arsenic**	**Selenium**	**Antimony**	**Arsenic**	**Selenium**	**Antimony**
GW1^*^	0	3.67±2.1	0	0	38.7±5.9	38.0±6.2
GW2^*^	0	3.33±2.1	15.0±6.2	0	38.0±5.3	41.0±6.2
GW3^*^	0	2.67±3.1	0	0	23.0±2.6	51.7±15
GW4^*^	0	10.7±7.6	8.0±7.0	13.3±6.8	46.7±9.3	49.7±15.2
GW5^*^	0	3.67±2.1	3.67±6.4	0	29.6±5.1	40.0±4.0
GW6^*^	0	4.33±1.5	0	4.33±4.0	18.3±2.5	57.7±5.0
GW7^*^	0	3.0±2.6	0	0	14.0±3.0	27.0±5.0
GW 8^*^	0	2.0±2.6	0	0	12.6±2.1	39.3±20
GW 9^*^	0	33.3±8.7	0	0	56.0±15.6	41.6±10.7
GW 10^*^	0	29.7±4.9	0	0	33.7±2.1	37.7±14.6
GW 11^*^	0	12.3±4.5	8.0±7.0	0	28.7±3.8	48.7±14.8
GW 12^*^	0	7.33±3.5	0	0	53.3±5.0	66.3±9.5
GW 13^*^	0	8.0±5.3	0	0	20.7±3.2	55.0±8.5
GW 14^*^	0	11.3±2.1	0	0	21.6±3.5	30.3±8.5
GW 15^*^	0	20.3±3.1	11.7±5.9	0	37.6±4.7	54.0±2.6
GW 16^*^	0	6.0±5.3	7.33±6.4	13.3±2.5	12.3±3.2	70.0±7.9
GW 17^*^	0	4.0±4.0	3.67±4.0	0	11.7±2.1	44.3±8.6
GW 18^*^	0	26.7±5.5	0	0	66.0±8.2	40.3±11.9
GW 19^*^	0	6.67±4.2	0	0	17.0±2.6	27.0±3.6
GW 20^*^	0	11.3±5.0	0	0	29.0±6.1	46.3±9.3
GW 21^*^	0	19.7±4.0	0	0	55.3±8.5	39.0±14
GW 22^*^	0	20.7±3.2	0	0	62.3±5.5	58.3±4.7
GW 23^*^	0	9.33±6.0	0	0	31.0±4.0	59.7±4.2
GW 24^*^	0	17.0±1.0	0	0	42.3±1.5	58.0±4.6
GW 25^**^	0	26.7±8.9	0	67.6±4.7	36.0±4.4	56.7±4.7
GW 26^**^	0	35.3±2.1	0	50.7±7.1	53.7±5.9	57.0±6.1
GW 27^**^	0	9.0±2.0	0	0	40.0±7.9	36.3±8.6
GW 28^**^	0	7.67±3.1	0	0	43.7±9.5	52.7±7.4
GW 29^**^	0	12.3±4.5	0	0	42.3±10.3	35.7±6.4
GW 30^***^	0	1.33±2.3	0	0	17.7±5.0	52.7±1.2
GW 31^***^	0	5.67±4.0	0	0	27.3±4.9	53.6±3.2
GW 32^***^	0	6.33±5.7	0	13.3±6.8	0.026	60.6±4.9
GW 33^***^	0	3.67±6.4	0	0	18.7±6.7	48.3±3.2
GW 34^****^	0	23.67±6.7	0	0	22.7±4.9	46.7±3.8
GW 35^****^	0	18.0±4.4	0	0	19.0±7.2	61.6±5.1

^*^Residential areas

^**^Commercial areas

^***^Industrial areas

^****^Agricultural areas

### Risk Factor for Arsenic, Antimony and Selenium in Groundwater

The calculated ADD, HQ and HI values from arsenic, antimony and selenium exposure in groundwater from the four studied areas is shown in Table 4. Average daily dose intakes of arsenic, antimony and selenium ranged between 0.000 to 0.001228 for adults, and 0.000 to 0.001535 for children. The HQ for antimony of 2.38 to 2.71 for adults and 2.975 to 3.3875 for children were significantly higher than for other metals. The hazard index of the three metals within the commercial areas was higher than the other areas under study. The adult population recorded an HQ of 4.1989, while the children's population had an HQ of 5.2487.

**Table 4:** Risk Assessment of Arsenic, Antimony and Selenium Exposure in Groundwater

**Sample Areas**	**Metals**	**C(mg/L)**	**Adults**				**Children**			
			**ADD (mg/kg/day)**	**RfD (mg/kg/day)**	**HQ**	**HI**	**ADD (mg/kg/day)**	**RfD (mg/kg/day)**	**HQ**	**HI**
Residential	Arsenic	0.00065	0.000026	0.0003	0.0867	2.7159	0.0000325	0.0003	0.1083	3.3948
	Antimony	0.0245	0.00098	0.0004	2.45		0.001225	0.0004	3.0625	
	Selenium	0.0224	0.000896	0.005	0.1792		0.00112	0.005	0.224	
Commercial	Arsenic	0.0118	0.000472	0.0003	1.5733	4.1989	0.00059	0.0003	1.9667	5.2487
	Antimony	0.0238	0.000952	0.0004	2.38		0.00119	0.0004	2.975	
	Selenium	0.0307	0.001228	0.005	0.2456		0.001535	0.005	0.307	
Industrial	Arsenic	0.00167	0.0000668	0.0003	0.2227	3.0199	0.0000835	0.0003	0.2783	3.7748
	Antimony	0.0269	0.001076	0.0004	2.69		0.001345	0.0004	3.3625	
	Selenium	0.0134	0.000536	0.005	0.1072		0.00067	0.005	0.134	
Agricultural	Arsenic	0.000	0.0000	0.0003	0.0000	2.8772	0.0000	0.0003	0.0000	3.5965
	Antimony	0.0271	0.001084	0.0004	2.71		0.001355	0.0004	3.3875	
	Selenium	0.0209	0.000836	0.005	0.1672		0.001045	0.005	0.209	

Abbreviations: ADD-Average daily dose; RfD, Reference dose

## Discussion

### Arsenic, Antimony and Selenium Contamination of Groundwater

Average arsenic levels were well below the WHO^23^ limit of 10 μg/L, while during the dry season period, <6% samples had very high arsenic levels exceeding the WHO limit.^23^ The locations with very high arsenic levels were largely characterized by heavy commercial activities associated with building material and automobile parts distribution. Evidence of major engine oil spillage, scrap metal parts and broken building materials were found in soils at these locations. Leaching and surface runoff may explain the high levels of arsenic in shallow groundwater obtained from these locations. Background concentrations of arsenic in groundwater in most countries are usually substantially lower than 10 μg/L. However, values quoted in the literature show a very wide range, from < 0.5 to 500 μg/L.^25^ Average arsenic concentrations in groundwater within the Ibadan metropolis were well below the levels of 552 and 353 μg/L reported in the Kandal Province of Cambodia,^1^ which has a similar urban structure to Ibadan metropolis. Previous studies have reported similar levels of arsenic (10–70 μg/L) in groundwater samples within the Odeda region of Ogun State, Nigeria.^57^ In comparison, there were no significant differences in seasonal variations in levels of arsenic in groundwater within the study period. This indicates little or no significant presence of arsenic in the groundwater in Ibadan metropolis. The few locations where arsenic was found could be regarded as outliers or localized contamination points.

A greater percentage (>70%) of the sampled wells recorded average antimony levels slightly higher than the safe limit of 20 μg/L for drinking water set by the WHO.^23^ These wells were sampled throughout residential, commercial, industrial and agricultural areas. A rather unique seasonal trend was observed for antimony in groundwater. Antimony was seldomly detected during the wet season, but levels up to 100% in excess of safe limits were recorded in all the studied wells during the dry season. There was a significant difference in seasonal antimony levels in groundwater. Low precipitation characterized by concentrated groundwater obtained during the dry period many explain these relatively high levels. Since groundwater occurs within geological formations, levels of antimony in the groundwater reserve may be caused by antimony from natural geological sources. It is also possible that infiltration of antimony arising from anthropogenic activities contributes to antimony levels in groundwater. The source(s) of antimony in groundwater cannot be specifically identified due to the absence of data on both antimony levels in the geological rock samples and surface soil around the Ibadan metropolis. Antimony in drinking water in Nigeria is attracting attention for a number of reasons. First, it is a new and unfamiliar problem for the general population and concerned professionals, and second, there are no data on its occurrence and distribution. There is concern that consumption of antimony rich water could cause adverse health effects either at present or in the future.

Selenium is an essential element, but is toxic at elevated concentrations in groundwater. Nutrition is the main source of selenium exposure to the general population.^58^ Selenium levels in drinking water are generally very low. In the present study, only 11.4% of samples had average concentrations slightly above the WHO^23^ limit of 40 μg/L. Selenium was well detected in all groundwater samples during both the wet and dry season periods. The dry season period recorded about 31.4% samples above the 40 μg/L limit. These locations were mostly within the residential and commercial areas of the metropolis. No significant differences in selenium levels between the wet and dry season were found, with a strong Pearson correlation index of 0.6125. The selenium level of <0.15 ng/mL determined in groundwater in Poland in a previous study was below average levels obtained in the present study.^25^ The average range in the current study was also below toxic concentrations of 45–341 μg/L recorded in groundwater from the Jainpur and Barwa villages in the states of Punjab and Haryana in northwest India,^59^ and well below the level of 0.17 to 0.44 ng/ml recorded in Poznan, Poland.^60^

The correspondence analysis relay plot shows the order of occurrence of these trace metals in groundwater to be antimony > selenium > arsenic. That is, antimony was more prevalent in groundwater followed by selenium and lastly arsenic. Principal component analysis (correlation matrix) found that two components accounted for 46.6% and 32.1% of total trace metal loads in groundwater (*Figure 2*). The first component is a size variable indicating that 45.7% of the sampling locations accounted for 46.6% of the total arsenic, antimony and selenium levels in the groundwater. These are points to the right of the plot and are located within the residential, commercial, industrial and agricultural areas of the metropolis. Points to the left represent 54.3% of sampling locations with lower loads of arsenic, antimony and selenium, accounting for 32.1% of total metals in groundwater. The principal component analysis biplot shows that arsenic, antimony and selenium were fairly distributed in the study area, suggesting the influence of geogenic factors. Analysis of variance showed a significant difference in average arsenic, antimony and selenium distribution within the study area. Groundwater antimony levels were significantly higher than the WHO^23^ standard in residential, commercial, industrial and agricultural areas, while arsenic levels were higher in the commercial area only, and selenium levels were well below defined standards (*Figure 3*).

**Figure 2:**
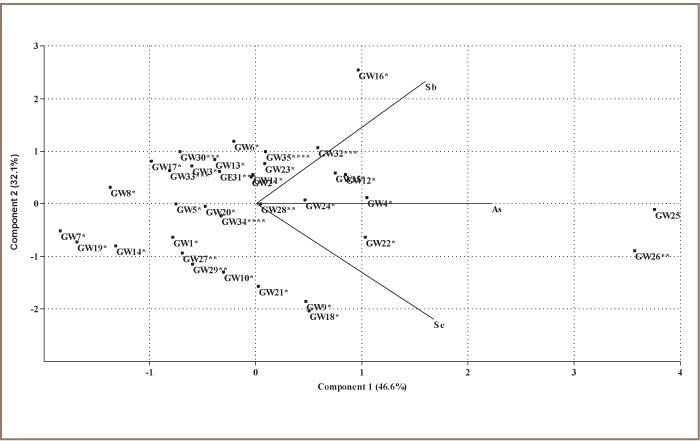
Principal component analysis biplot of trace metal levels in groundwater

**Figure 2:**
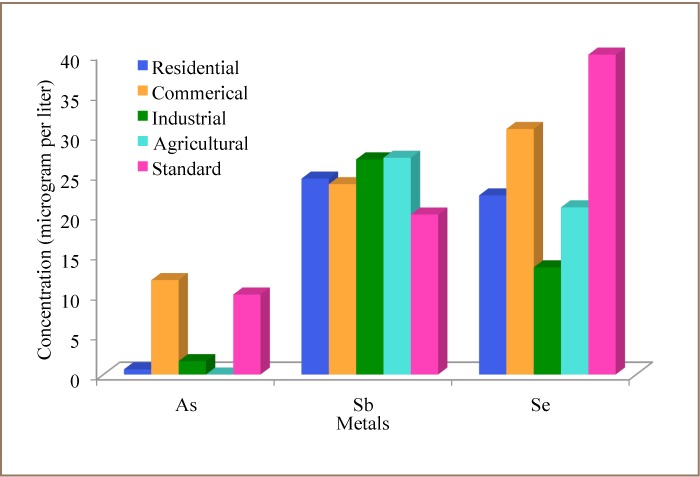
Principal component analysis biplot of trace metal levels in groundwater

### Risk Assessment of Arsenic, Antimony and Selenium in Groundwater

The average daily dose intake of arsenic, antimony and selenium combined in groundwater was similar across the studied areas for adults and children. Both age groups had antimony and selenium daily dose intake levels of about 0.001 mg/kg/day, which was higher than for arsenic. The chemical of greatest potential human health concern was antimony, with a non-carcinogenic HQ risk factor of >2 for both age groups. The hazard quotient cannot be translated into a probability that adverse health effects will occur, and is unlikely to be proportional to risk. Therefore, an HQ value exceeding 2 does not necessarily mean that adverse effects will occur. The overall non-carcinogenic HI derived for arsenic, antimony and selenium showed that the commercial area had the highest values for adults and children. Antimony in drinking water occasionally generates concern due to its potential human health risk, and children in the commercial setting had the highest levels of exposure by body weight within the metropolis. The hazard quotient of non-carcinogen arsenic in ground water in Ibadan metropolis was similar to that calculated for an unconsolidated shallow ground water aquifer in the Midyan Basin of northwestern Saudi Arabia for adults and children.^51^

## Conclusions

The present study revealed slightly elevated groundwater concentrations of antimony within the Ibadan metropolis. A total of 74.3% of sampled groundwater wells contained antimony concentrations above the WHO safe drinking water limit of 20 μg/L. In addition, antimony was found to be the dominant contaminant in groundwater in the dry season, with 100% of samples exceeding WHO guidelines. These antimony concentrations were fairly distributed throughout residential, commercial, industrial and agricultural areas of the city metropolis, consistent with the influence of either geogenic or anthropogenic factors. There was little or no significant presence of arsenic or selenium in groundwater wells in the metropolis. Antimony was the chemical of greatest potential human health concern with a non-carcinogenic HQ risk factor >2 for both age groups. The over-dependence on groundwater for drinking water could pose a great health risk to residents of the metropolis, putting millions of people at serious risk of antimony poisoning, especially during the dry season. Further studies on the chemistry of the local geological rock formations and soil need to be carried out to establish the potential source of antimony in the groundwater within Ibadan metropolis.
